# Whole-Genome Analysis of Candidate genes Associated with Seed Size and Weight in *Sorghum bicolor* Reveals Signatures of Artificial Selection and Insights into Parallel Domestication in Cereal Crops

**DOI:** 10.3389/fpls.2017.01237

**Published:** 2017-07-18

**Authors:** Yongfu Tao, Emma S. Mace, Shuaishuai Tai, Alan Cruickshank, Bradley C. Campbell, Xianrong Zhao, Erik J. Van Oosterom, Ian D. Godwin, Jose R. Botella, David R. Jordan

**Affiliations:** ^1^Queensland Alliance for Agriculture and Food Innovation, University of Queensland Warwick, QLD, Australia; ^2^Department of Agriculture and Fisheries, Hermitage Research Facility Warwick, QLD, Australia; ^3^BGI Genomics, BGI-Shenzhen Shenzhen, China; ^4^School of Agriculture and Food Sciences, University of Queensland Brisbane, QLD, Australia; ^5^Queensland Alliance for Agriculture and Food Innovation, University of Queensland Brisbane, QLD, Australia

**Keywords:** sorghum, seed size, orthologs, comparative genomics, selection signatures, domestication

## Abstract

Seed size and seed weight are major quality attributes and important determinants of yield that have been strongly selected for during crop domestication. Limited information is available about the genetic control and genes associated with seed size and weight in sorghum. This study identified sorghum orthologs of genes with proven effects on seed size and weight in other plant species and searched for evidence of selection during domestication by utilizing resequencing data from a diversity panel. In total, 114 seed size candidate genes were identified in sorghum, 63 of which exhibited signals of purifying selection during domestication. A significant number of these genes also had domestication signatures in maize and rice, consistent with the parallel domestication of seed size in cereals. Seed size candidate genes that exhibited differentially high expression levels in seed were also found more likely to be under selection during domestication, supporting the hypothesis that modification to seed size during domestication preferentially targeted genes for intrinsic seed size rather than genes associated with physiological factors involved in the carbohydrate supply and transport. Our results provide improved understanding of the complex genetic control of seed size and weight and the impact of domestication on these genes.

## Introduction

A growing world population and an increase in affluence is driving demand for agricultural products, especially cereals, which supply more than 75% of the calories consumed by humans (Sands et al., 2009). With limited arable land and water resources, particularly in Sub-Saharan Africa where sorghum is a staple food and the population growth rate is amongst the highest in the world, enhancing yield per unit area of cereal crops will be critical to meet this demand. Seed number per unit area and seed size are critical components of seed yield. Although seed number tends to have a bigger influence on yield (Boyles et al., 2016), seed size can make a significant contribution and may offer prospects for further yield improvement (Yang et al., 2009). In addition, it is often a major quality attribute (Lee et al., 2002). Hence, elucidating the genetic basis of seed size and the impact of domestication on seed size genes in sorghum will enhance the understanding of crop domestication and provide new targets for manipulating seed size in breedingpractice.

Seed size is an important fitness trait for flowering plants and plays an important role in adaptation to particular environments. Under natural conditions, greater seed resources stored in larger seeds enable seedlings to grow more rapidly at the seedling stage and increases competitiveness and survival (Manga and Yadav, 1995). However, increased seed number also translates directly into fitness, resulting in selection pressure to produce more (and thus smaller) seeds (Westoby et al., 1992). For cereal crops, the preference of early farmers for large seeded lines for easier harvesting, processing, and planting has resulted in larger seed size being selected during domestication. This selection process has left observable genetic changes, including a reduction of genetic diversity and an increased frequency of favorable seed size alleles in cultivated lines compared to their wild progenitors (Doebley et al., 2006). For example, in rice, the favorable allele of *GS3*, which encodes a heterotrimeric G-protein subunit that affects seed weight and length, was highly enriched in a set of cultivated accessions of rice (*Oryza sativa* L.) (34%) compared to a set of wild accessions (4%; Takano-Kai et al., 2009; Botella, 2012). In maize (*Zea mays* L.), *Bt2*, which encodes the small subunit of the ADP-glucose pyrophosphorylase involved in starch biosynthesis and seed weight, has shown a 3.9-fold reduction in genetic diversity in cultivated inbred lines compared to their wild teosinte relatives (Whitt et al., 2002). Likewise, selection signatures have also been identified on other seed size genes, including *PBF1* (Lang et al., 2014), *GS5* (Li et al., 2011), and *GIF1* (Wang et al., 2008). These selection signatures provide a “bottom-up” approach to investigate the genetic basis of domesticated traits, which has been successfully implemented in many species for other traits such as prolificacy (Beissinger et al., 2014) and northern leaf blight resistance (Wisser et al., 2008) in maize.

Seed size is a physiologically complex trait. Sorghum seeds are typically tending toward spherical, although considerable phenotypic variation in length, width and density does exist. The potential size of the seed is often associated with cell number, cell size and number of starch granules and is highly correlated with ovary volume at anthesis (Yang et al., 2009). However, measures associated with seed size have not been used consistently in the literature, where individual grain weight is often used as a surrogate for seed size. As key components of carbon demand (sink) during seed filling, seed size and weight are strongly associated with both carbon supply (source) and transport between carbon sources and the seed (path). The potential mass of individual seeds is determined by the rate and duration of seed filling. In sorghum, seed filling rate is highly correlated with ovary volume at anthesis, which in turn is associated with the size of the meristematic dome during early floret development (Yang et al., 2009).

Although seeds with larger potential size tend to have greater seed mass, the extent to which this increased seed mass is actually achieved is strongly determined by assimilate availability for each seed. The amount of assimilate per seed is driven by factors affecting both seed number and assimilate supply. Total seed number per plant is determined by the number of seeds per panicle and the number of panicles per plant (i.e., tillering and branching), which are affected by a range of genetic and environmental factors (Alam et al., 2014). A negative correlation between seed size and seed number has been observed frequently in cereals (Jakobsson and Eriksson, 2000; Acreche and Slafer, 2006; Peltonen-Sainio et al., 2007; Sadras, 2007). Specifically in sorghum this trade-off has been observed by different groups (Heinrich et al., 1983; Yang et al., 2010; Burow et al., 2014). Traits such as number of seeds per panicle and number of tillers per plant are also commonly negatively correlated with seed size (Moles and Westoby, 2004). Contributors of assimilate availability for seed filling, including photosynthesis (Jagadish et al., 2015), have shown positive correlations with seed size. Environmental factors can also exert a strong influence on seed size by affecting assimilate supply (Jenner, 1994; Borrell et al., 2014) and carbon translocation(Zolkevich et al., 1958).

In accordance with this physiological complexity, seed size has been identified as a quantitative trait controlled by multiple genes, many of which have been cloned in model species (Xing and Zhang, 2010; Li et al., 2013; Zuo and Li, 2014). In *Arabidopsis*, a kinase cascade consisting of *HAIKU1, HAIKU2*, and *MINISEED3* promotes seed development zygotically (Luo et al., 2005; Wang et al., 2010), while *TTG2* (Garcia et al., 2005), *AP2* (Ohto et al., 2009), and *ARF2* (Okushima et al., 2005) are engaged in the maternal control of seed size. In rice, QTLs including *GS3* (Mao et al., 2010), *GS5* (Li et al., 2011), *GW2* (Song et al., 2007), *GW5* (Liu et al., 2017), *GW8* (Wang S. et al., 2012), and *GL7* (Wang Y. et al., 2015) were reported to regulate seed size by controlling cell division, while the influence of *SRS3* (Kitagawa et al., 2010), *D61* (Morinaka et al., 2006), and *SRS5* (Segami et al., 2012) on seed size is related to the regulation of cell size. Additionally, the role of *GIF1* in carbon partitioning during early seed-filling, which can impact seed weight, has been identified using functional analysis in rice (Wang et al., 2008). In maize, the *Gln-4* gene (Martin et al., 2006) affects seed weight by controlling nitrogen transport to the kernel during seed-filling, whereas *Sh2*, which encodes the large subunit of ADP-glucose pyrophosphorylase, affects seed weight by regulating starch biosynthesis (Jiang L. et al., 2013). Pleiotropy is common amongst genes affecting seed size. For example, *D2* (Hong et al., 2003) and *SMG1* (Duan et al., 2014) also have an effect on plant architecture, *TH1* (Li X. et al., 2012) affects seed number, and *TGW6* (Ishimaru et al., 2013) influences translocation efficiency from source organs. These genes may thus affect seed size via source-sink dynamics.

Sorghum, second only to maize among C_4_ cereals in terms of the scale of grain production, is known for its adaptation to heat and drought stress, and is a staple for 500 million of the world's poorest people. Despite the great importance of this crop, the genetic basis of seed size in sorghum has been the subject of relatively few studies and little information is available about genetic control of the trait and signatures of domestication. Hence, this study aims to investigate the polymorphism patterns and signatures of domestication of candidate genes associated with seed size and weight by using resequencing data for a diverse group of wild and weedy and landrace genotypes (Mace et al., 2013) in order to enhance understanding of crop domestication and to provide potential targets for manipulating seed size in sorghum breeding.

## Materials and methods

### Data collection

Genes associated with seed size and weight (hereafter referred as seed size) in three species, maize, rice and *Arabidopsis*, were identified through a comprehensive literature review (Table S1). Seed length, seed width, and seed density are all potentially associated with seed size; therefore multiple parameters including thousand seed weight, seed length, and seed width, were used as keywords for literature searches. A subset of high confidence genes were identified with evidence of their association with seed size supported by QTL cloning, transgenic experiments, mutant analysis, association signal, and/or near isogenic lines analysis.

Genome assemblies and predicted gene models and protein sequences for *Arabidopsis thaliana* (TAIR10), *Oryza sativa* (IRGSP-1.0), *Zea mays* (AGPv4), and *Sorghum bicolor* (v3.0) were downloaded from TAIR (https://www.arabidopsis.org); The Rice Annotation Project database (http://rapdb.dna.affrc.go.jp); Gramene (http://www.gramene.org) and Joint Genome institute (http://www.phytozome.net), respectively.

### Identification of orthologos genes

Orthologous genes in sorghum were identified by combining synteny-based and the Bidirectional Best Hit (BBH) approaches (Wolf and Koonin, 2012). Genomic syntenic relationships between sorghum and model species were extracted from Plant Genome Duplication Database (http://chibba.agtec.uga.edu/duplication/) and used to search for syntenic orthologs, while a local BLAST strategy was used for the BBH approach to identify pairs of genes in two genomes that are the best BLAST hits (highest score) to one another, using BLASTP.

### Expression analysis of seed size candidate genes

The whole genome expression data from the study by Davidson et al. (2012) was used to investigate the differential expression of the 114 candidate genes. The data set compared expression of genes in the seed at two different time points and two different seed tissues in addition to five non-seed tissues (Davidson et al., 2012). The maximum expression value (Fragments Per Kilobase of transcript per Million mapped reads, FPKM) from any of the seed tissue samples was compared to the maximum expression value in any of the non-seed tissues and a fold difference >2 was used to define genes that were differentially highly expressed in the seed.

### Population genetics analysis

#### Gene level population genetics parameters

The sequence data of the seed size genes in sorghum were extracted from the whole genome resequencing data as described in Mace et al. (2013) for 25 sorghum genotypes, representing two groups: (1) wild and weedy genotypes and (2) landraces. A number of summary statistics based on gene level, including the average pairwise genetic diversity within a group, θπ (Nei and Li, 1979) and Tajima's D (Tajima, 1989), were calculated using a BioPerl module and an in-house perl script. F_ST_ (Hudson et al., 1992) was calculated to measure population differentiation using another BioPerl module. Reduction of diversity (RoD) during domestication was calculated as fold of decrease of θπ in the landrace group compared to the wild and weedy group.

#### Identifying selection signatures at the SNP level

CDS of the seed size genes across 25 resequenced genotypes was used to generate population statistics for every SNP using the R package PopGenome (Pfeifer et al., 2014). Specifically, a 1-bp window size with a 1-bp step size was used to define the slide window. θπ (Nei and Li, 1979), Fst (Hudson et al., 1992), and Tajima's D (Tajima, 1989) for each SNP within the CDS were calculated using diversity.stats, F_ST.stats, and neutrality.stats commands. Functional information was estimated by get.codons. RoD in the pairwise ancestor/descendant population comparison was calculated as fold of decrease of θπ in landrace compared to wild and weedy. To identify SNPs under purifying selection the following criteria were used: (1) RoD in the pairwise ancestor/descendant population comparison should be greater than the average RoD based on 159 neutral loci; (2) F_ST_ should be positive; (3) Tajima's D should be negative.

#### mlHKA test

A set of 63 seed size candidate genes under purifying selection were used as input, together with three random selections of 36 genes from 159 neutral genes, for the mlHKA (Wright and Charlesworth, 2004) test for validation purposes. The mlHKA program was run under a neutral model, where numselectedloci = 0, and then under a selection model, where numselectedloci >0. The number of cycles of the Markov chain was set to be 100,000. For each random selection of 36 neutral genes, three random numbers of seed were set to be 10, 20, and 30, respectively. This means 3 × 3 = 9 times of run were performed. Significance was assessed by the mean log likelihood ratio test statistic, where twice the difference in log likelihood between the models is approximately chi-squared distributed with df equal to the difference in the number of parameters.

#### Haplotype analysis of genes under selection

Haplotype analysis was performed using R package pegas (Population and Evolutionary Genetics Analysis System; Paradis, 2010) and ape package (Paradis et al., 2004) for genes under selection. Functions haplotype, haploFreq and haploNet were called to generate haplotype maps. In addition to landrace and wild & weedy, accessions from improved lines, *Guinea margaritiferum* race and *S. propinquum* were used in haplotype analyses (Table S2).

## Results

### Seed size candidate genes in sorghum

**Figure 1:**
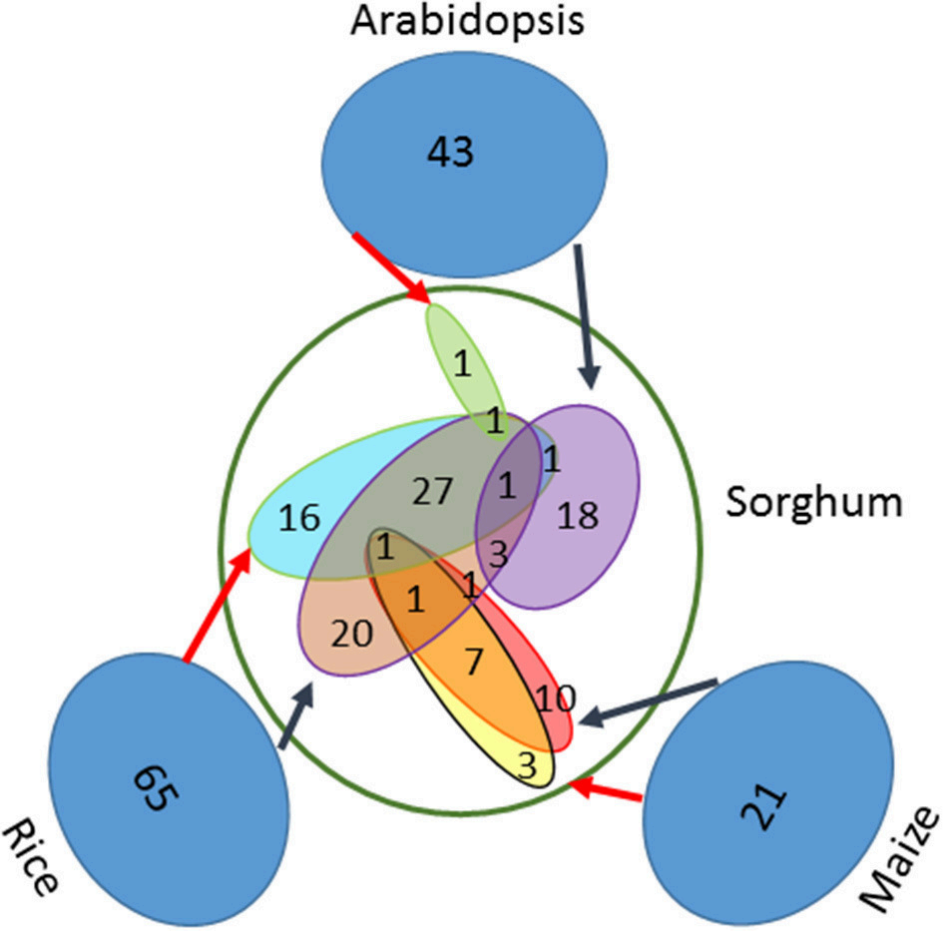
One hundred eleven orthologs of seed size genes identified in sorghum. Both the BBH method and the known syntenic relationships were used to identify orthologs of previously identified seed size genes in Arabidopsis (43), maize (21), and rice (65). The black arrows indicate BBH-identified orthologs, while the red arrows indicate syntenic orthologs.

Based on a comprehensive literature survey, 129 genes associated with seed size were identified in three well-studied model species, including 65 genes in rice, 21 in maize and 43 in *Arabidopsis* (Table S1). By using BBH method and the known syntenic relationship from the Plant Genome Duplication Database to infer orthologs (assembly v3.0), a total of 111 genes were identified in sorghum (Table 1). From the set of 65 seed size-related genes identified in rice, 55 orthologs were identified in sorghum using the BBH method and 47 using the syntenic relationship method. Of these, 30 orthologs were identified by both methods, resulting in a total of 72 unique orthologs identified in sorghum (Figure 1). Additionally, a total of 23 orthologs were identified in sorghum based on the 21 seed size-related genes from maize, including 20 BBH orthologs and 12 syntenic orthologs with 9 orthologs identified by both methods. Finally, 25 sorghum orthologs were identified based on the analysis of the 43 selected seed size-related genes from *Arabidopsis* (Figure 1). Amongst all putative sorghum orthologs, 9 were in common across a minimum of two species, leading to 111 unique orthologs in sorghum identified as seed size candidate genes (Figure 1). Four seed size candidate genes in sorghum from Zhang et al. (2015) with one overlapped with the 111 seed size orthologs were also taken into consideration, resulting in a final list of 114 seed size candidate genes.

**Table 1:** Seed size candidate genes identified in sorghum including details of the identification approach, the original study describing the gene's function and presence of supporting selection.

**Gene ID^a^**	**Approach^b^**	**Original gene^c^**	**Selection signature^d^**	**References^e^**
Sobic.001G016200	BBH	*Nuf2 family protein*	Yes	Huang et al., 2012b
Sobic.001G056700	Synteny	*O2*	Yes	Hartings et al., 1989
Sobic.001G107100	BBH	*SRS5*	Yes	Segami et al., 2012
Sobic.001G113200	BBH	AHK4	Yes	Riefler et al., 2006
Sobic.001G154900	Both	GL3.1/qGL3	Yes	Qi et al., 2012; Zhang et al., 2012
Sobic.001G170800	Both	Transport protein	Yes	Huang et al., 2012b
Sobic.001G172400	BBH	BRD1	Yes	Mori et al., 2002
Sobic.001G184900	Both	Expressed protein	Yes	Huang et al., 2012b
Sobic.001G254100	Synteny	*PGL1*	No	Heang and Sassa, 2012a
Sobic.001G254200	Synteny	OsFBK12	Yes	Chen et al., 2013
Sobic.001G285000	BBH	IKU1	No	Wang et al., 2010
Sobic.001G335800	Synteny	qGW7/GL7	Yes	Wang S. et al., 2015; Wang Y. et al., 2015
Sobic.001G336200	BBH/BBH	KLU/ Grain Length3.2	No	Adamski et al., 2009; Xu et al., 2015
Sobic.001G341700	BBH/Both	GS3/ZmGS3	Yes	Li et al., 2010b; Mao et al., 2010
Sobic.001G382400	BBH	FER	No	Yu et al., 2014
Sobic.001G445900	BBH/BBH	CYP90B2/CYP90B1	No	Wu et al., 2008
Sobic.001G448700	Both	TUD1	No	Hu et al., 2013
Sobic.001G468400	Both	Prol1.1	No	Wills et al., 2013
Sobic.001G482600	BBH	TIFY 11b	No	Hakata et al., 2012
Sobic.001G484200	BBH	RGA1/D1	No	Ashikari et al., 1999
Sobic.001G485400	Both	BG1	No	Liu L. et al., 2015
Sobic.001G488400	Synteny	*PGL1*	No	Heang and Sassa, 2012a
Sobic.001G488500	BBH	OsFBK12	No	Chen et al., 2013
Sobic.002G021200	BBH	DDM1	Yes	Xiao et al., 2006
Sobic.002G022600	BBH	ANT	No	Mizukami and Fischer, 2000
Sobic.002G054800	Both	*O2*	Yes	Hartings et al., 1989
Sobic.002G056000	BBH	MET1	Yes	Xiao et al., 2006
Sobic.002G116000	BBH	GbssIIa	No	Jiang L. et al., 2013
Sobic.002G216600	Both/Synteny	DEP1/AGG3	No	Huang et al., 2009; Li S. et al., 2012
Sobic.002G226500	Both	SG1	Yes	Nakagawa et al., 2012
Sobic.002G257900	Synteny	GW8	Yes	Wang S. et al., 2012
Sobic.002G272700	BBH	EOD3/CYP78A6	Yes	Fang et al., 2012
Sobic.002G308400	BBH	MYB transcription factor	Yes	Huang et al., 2012b
Sobic.002G309600	BBH	UPF1	Yes	Yoine et al., 2006
Sobic.002G311000	BBH	Receptor-like kinase	Yes	Huang et al., 2012b
Sobic.002G312200	Both	GLW7	No	Si et al., 2016
Sobic.002G367300	Both	qGW7/GL7	Yes	Wang S. et al., 2015; Wang Y. et al., 2015
Sobic.002G367600	BBH	BG2	No	Xu et al., 2015
Sobic.002G374400	Both	DEP2	Yes	Li F. et al., 2010
Sobic.003G014500	BBH	MHZ7	No	Ma et al., 2013
Sobic.003G030600	BBH	*D2*	No	Hong et al., 2003
Sobic.003G035400	Synteny	GW5/qSW5	No	Liu et al., 2017
Sobic.003G140000	Synteny	OsSAMS1	Yes	Chen et al., 2013
Sobic.003G230500	BBH	*Sh2*	Yes	Jiang L. et al., 2013
Sobic.003G277900	BBH/BBH	D61/ BRI1	Yes	Morinaka et al., 2006; Jiang W. et al., 2013
Sobic.003G292600	BBH	AHP4	No	Hutchison et al., 2006
Sobic.003G358400	BBH	DET2	No	Jiang W. et al., 2013
Sobic.003G380900	Synteny	*SERF1*	No	Schmidt et al., 2013
Sobic.003G406600	Synteny	GW8	Yes	Wang S. et al., 2012
Sobic.003G407300	BBH	AHK3	No	Riefler et al., 2006
Sobic.003G444100	Both	OsCCS52B	Yes	Su'udi et al., 2012
Sobic.004G065400	Synteny	*GW6*	No	Song et al., 2015
Sobic.004G075600	Both	Zinc finger protein	Yes	Huang et al., 2012b
Sobic.004G085100	Both	Bt1	Yes	Shannon et al., 1998
Sobic.004G093900	BBH	CKX2	Yes	Li et al., 2013
Sobic.004G107300	Both/Both/Both	GW2/ZmGW2-4/ZmGW2-5	No	Song et al., 2007; Li et al., 2010a
Sobic.004G133600	BBH/original	ZmSWEET4c/NA	No	Sosso et al., 2015; Zhang et al., 2015
Sobic.004G163700	BBH	SbeIIb	No	Jiang L. et al., 2013
Sobic.004G176000	Both	SDG725	Yes	Sui et al., 2012
Sobic.004G214100	Both	BC14	No	Zhang et al., 2011
Sobic.004G237000	Both	*PGL2*	No	Heang and Sassa, 2012b
Sobic.004G245000	Synteny	AHK4	Yes	Riefler et al., 2006
Sobic.004G247000	Both	*Gln-4*	No	Martin et al., 2006
Sobic.004G269900	Synteny	GS2/GL2	Yes	Che et al., 2015; Hu et al., 2015
Sobic.004G307800	Both	SGL1	Yes	Nakagawa et al., 2012
Sobic.004G317300	BBH	O1	Yes	Wang G. et al., 2012
Sobic.004G323600	Both	*SMG1*	No	Duan et al., 2014
Sobic.004G330200	BBH	*TGW6*	Yes	Ishimaru et al., 2013
Sobic.004G338400	BBH	*TH1*	No	Li X. et al., 2012
Sobic.005G001500	Both	*PBF1*	Yes	Lang et al., 2014
Sobic.005G132000	BBH	*ARF2*	Yes	Okushima et al., 2005
Sobic.006G059900	Both	*ZmIPT2*	Yes	Weng et al., 2013
Sobic.006G080500	BBH	RGE1	Yes	Kondou et al., 2008
Sobic.006G114600	BBH/BBH	D11/CYP724B3	Yes	Tanabe et al., 2005; Wu et al., 2008
Sobic.006G203400	Synteny	GS2/GL2	Yes	Che et al., 2015; Hu et al., 2015
Sobic.006G239000	Both	FLO2	Yes	She et al., 2010
Sobic.006G240700	BBH	*AP2*	No	Ohto et al., 2009
Sobic.006G268800	Original	NA	Yes	Zhang et al., 2015
Sobic.007G032400	Both/BBH	OsFIE2/FIE	No	Luo et al., 2000; Na et al., 2012
Sobic.007G101500	BBH	*Bt2*	Yes	Jiang L. et al., 2013
Sobic.007G149200	Synteny/BBH	DEP1/AGG3	Yes	Huang et al., 2009; Li S. et al., 2012
Sobic.007G156800	Synteny	SGL1	No	Nakagawa et al., 2012
Sobic.007G166600	Original	NA	No	Zhang et al., 2015
Sobic.007G193500	Both	GW8	No	Wang S. et al., 2012
Sobic.008G001700	Synteny	*PBF1*	Yes	Lang et al., 2014
Sobic.008G100400	BBH	*SMK1*	Yes	Li et al., 2014
Sobic.008G152800	BBH	CBL3	No	Eckert et al., 2014
Sobic.008G173900	Both	OsPPKL3	Yes	Zhang et al., 2012
Sobic.008G193300	BBH	OsSUT2	No	Eom et al., 2011
Sobic.009G024600	Both	RSR1	No	Fu and Xue, 2010
Sobic.009G033600	Both	OsSAMS1	Yes	Chen et al., 2013
Sobic.009G036400	BBH	APG	No	Heang and Sassa, 2012b
Sobic.009G040700	Both	OsPPKL2	Yes	Zhang et al., 2012
Sobic.009G049400	Both	*SRS3*	Yes	Kitagawa et al., 2010
Sobic.009G053600	BBH	*GS5*	Yes	Li et al., 2011
Sobic.009G070000	Both	GW5/qSW5	Yes	Liu et al., 2017
Sobic.009G141500	Synteny	*SERF1*	No	Schmidt et al., 2013
Sobic.010G022600	BBH	Wx1	Yes	Shure et al., 1983
Sobic.010G047400	Both	*HGW*	Yes	Li J. et al., 2012
Sobic.010G064600	BBH	*DA1*	No	Li et al., 2008
Sobic.010G064800	BBH	CKI1	Yes	Deng et al., 2010
Sobic.010G069600	Synteny	*SMG1*	Yes	Duan et al., 2014
Sobic.010G072300	Both	*Sh1*	No	Jiang L. et al., 2013
Sobic.010G091700	Synteny	*PGL2*	No	Heang and Sassa, 2012b
Sobic.010G110100	BBH	A transcription factor	Yes	Huang et al., 2012b
Sobic.010G111200	BBH	GASR7	No	Huang et al., 2012b
Sobic.010G144900	Original	NA	No	Zhang et al., 2015
Sobic.010G184100	Synteny	Bt1	No	Shannon et al., 1998
Sobic.010G210100	Both	*GW6*	Yes	Song et al., 2015
Sobic.010G228100	Both	DEP3	Yes	Qiao et al., 2011
Sobic.010G273900	BBH	SbeI	No	Jiang L. et al., 2013
Sobic.010G277300	BBH	BRD2	No	Hong et al., 2005
Sobic.K041100	BBH	*GIF1*	Yes	Wang et al., 2008
Sobic.K041200	BBH	Mn1	Yes	Miller and Chourey, 1992

a *Based on sorghum genome assembly 3.0*.

b *Bioinformatics approach used to identify seed size candidate genes*.

c *Gene name from the original publication in either maize, rice, or Arabidopsis*.

d *Selection signature based on SNP level analysis*.

e *Publication documenting the genes associated with seed size*.

The 114 identified seed size candidate genes were unevenly distributed across the 10 sorghum chromosomes, ranging from 23 genes located on chromosome 1 to only 2 genes located on chromosome 5. Whole genome expression data from the study by Davidson et al. (2012) was used to investigate the differential expression of the 114 candidate genes. A total of 22 genes exhibited differentially high levels of expression in the seed (Table S3).

### Genetic diversity in seed size genes in sorghum

**Figure 2:**
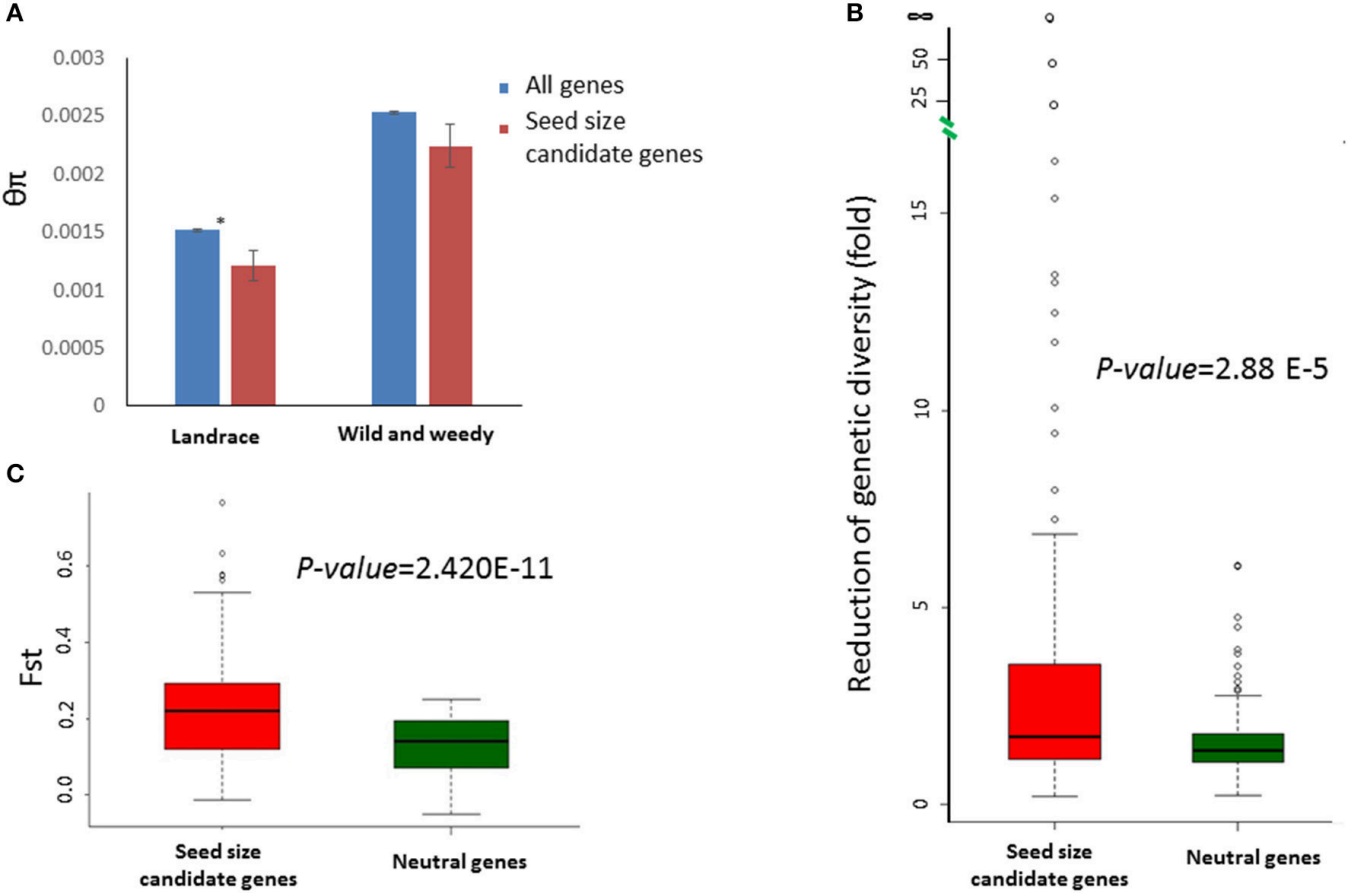
Sequence variation identified in the seed size candidate genes in sorghum. **(A)** A comparison of sequence diversity (θπ) between the seed size candidate genes (red) and genome-wide averages (blue) in both the landrace and wild and weedy groups. Error bars indicate the standard error; ^*^ indicates a significant difference (*p* < 0.05, *t*-test) between the groups. **(B)**, Box-plots showing the distributions of sequence diversity reduction fold) of 114 seed size candidate genes (red) and 159 neutral genes (blue) during domestication. The *p*-value was calculated based on a *t*-test. **(C)**, Box-plots showing the distributions of F_*ST*_ between the landrace and wild and weedy genotype groups for 114 seed size candidate genes (red) and 159 (blue) neutral genes. The *p*-value was calculated based on a *t*-test.

Sequence data for all 114 candidate genes was extracted from a previously described set of wild and weedy genotypes and landraces (Table S2; Mace et al., 2013). Overall, the selected genes exhibited a wide range of variation in sequence diversity in both genotype groups (the wild and weedy genotype group and the landraces group), with diversity measures (θπ) varying from 0.0085 (Sobic.002G311000) to 0 (Sobic.003G380900) in the wild and weedy genotypes, and from 0.0070 (Sobic.004G317300) to 0 (Sobic.003G035400, Sobic.003G380900, Sobic.004G065400, and Sobic.006G059900) in the landraces (Table S4). The *SERF1* (a negative regulator of seed filling in rice) ortholog, Sobic.003G380900, was invariant in all the genotypes included in the current study. The sequence diversity observed in the seed size candidate genes in the wild and weedy genotypes was not significantly different to the genome-wide averages. However, the seed size candidate genes in the landraces were significantly less diverse than the genome-wide averages (*p* = 0.026, *t*-test) (Figure 2A) and were significantly less diverse in comparison to the wild and weedy genotypes (*p* = 3.68E-11, paired *t*-test). The RoD in the seed size candidates between the two genotype groups during domestication was greater when compared to 159 neutral genes identified in a previous study (Mace et al., 2013; Table S5, Figure 2B). The degree of population differentiation, measured by the fixation index F_ST_, based on the seed size candidate genes was significantly higher between the landrace and wild and weedy genotypes (Figure 2C) in contrast to the neutral genes.

Furthermore, the extent of RoD varied among the seed size candidate genes. Two genes, Sobic.006G059900 (*ZmIPT2* ortholog) and Sobic.003G035400 (*GW5* ortholog), were invariant in the landrace genotypes, despite having high levels of sequence diversity in the wild and weedy genotypes. The signature of significantly reduced sequence diversity in the landrace group, in comparison to the wild and weedy group, was also observed in four other genes, with RoD ranging from 15- to 58-fold: Sobic.003G030600 (58-fold decrease), Sobic.003G277900 (25-fold decrease), Sobic.007G149200 (20-fold decrease), and Sobic.003G230500 (15-fold decrease). A contrasting signature of increased sequence diversity in the landraces was observed for 16 seed size candidate genes, including Sobic.004G237000, a syntenic ortholog of *PGL2*, with θπ of 0.0048 in the landrace genotypes in comparison to just 0.0021 in the wild and weedy genotypes. In addition to reduced sequence diversity in the landraces, a more skewed allele frequency, as determined through a negative Tajima's D value, was observed in the majority of cases.

### Signatures of selection in seed size candidate genes

Based on the genome-wide thresholds for the gene-level rankings described in Mace et al., (2013), 6 seed size candidate genes were identified with signatures of purifying selection during sorghum domestication (Table S6). Previous studies (Whitt et al., 2002; Brugiere et al., 2008; He et al., 2011; Hufford et al., 2012; Jiao et al., 2012; Xu et al., 2012; Luo et al., 2013; Weng et al., 2013; Wills et al., 2013; Lang et al., 2014; Zuo and Li, 2014; Sosso et al., 2015; Si et al., 2016) revealed purifying selection signals in 7 maize and 9 rice seed size genes included in this study (Table S1). Twenty one orthologs were identified in sorghum from 15 of the 16 genes under selection in either maize or rice, however, only one of them, Sobic.006G059900 (*ZmIPT2* ortholog), was identified with signatures of purifying selection in sorghum based on the gene-level rankings (Table S6).

To investigate the domestication signature in the 114 sorghum seed size candidate genes at a higher resolution, signatures of purifying selection at the SNP level were analyzed. In total, 2,317 SNPs were identified in the CDS of all 114 candidate genes, consisting of 1,202 synonymous SNPs and 1,115 non-synonymous SNPs. In addition to sequence diversity (θπ) metrics, F_ST_, Tajima's D, and RoD during domestication were calculated for each SNP. Based on the specified criteria regarding these metrics (see methods), 283 SNPs from 63 genes were identified with signatures of purifying selection, including Sobic.003G406600 (*GW8* ortholog), Sobic.008G100400 (*SMK1* ortholog), and Sobic.009G053600 (*GS5* ortholog). Out of the 63 genes under selection, 42 contained non-synonymous SNPs under selection (Table S7). The selection signatures identified at the SNP level included 5 out of 6 genes under selection at the gene level.

To validate whether the 63 selection candidates displayed patterns of genetic variation consistent with purifying selection, the mlHKA test was employed. A model of directional selection best explained the patterns of polymorphism observed relative to 159 neutral loci (mean log likelihood ratio test statistic = 661, *P* < 7.49E-94 for all comparisons, Table S8). Additionally, out of 22 seed size candidates exhibiting differentially high levels of expression in the seed, 17 (77%) were under selection. The percentage is significantly higher than the remaining 92 seed size genes not exhibiting differentially higher levels of expression in the seed, where only 46 genes (50%) in this group were found to be under selection (χ^2^ = 6.546, *p*-value < 0.05), indicating seed size genes highly expressed in the seed are more likely to be targeted during domestication.

### Parallel domestication of seed size in cereals

**Figure 3:**
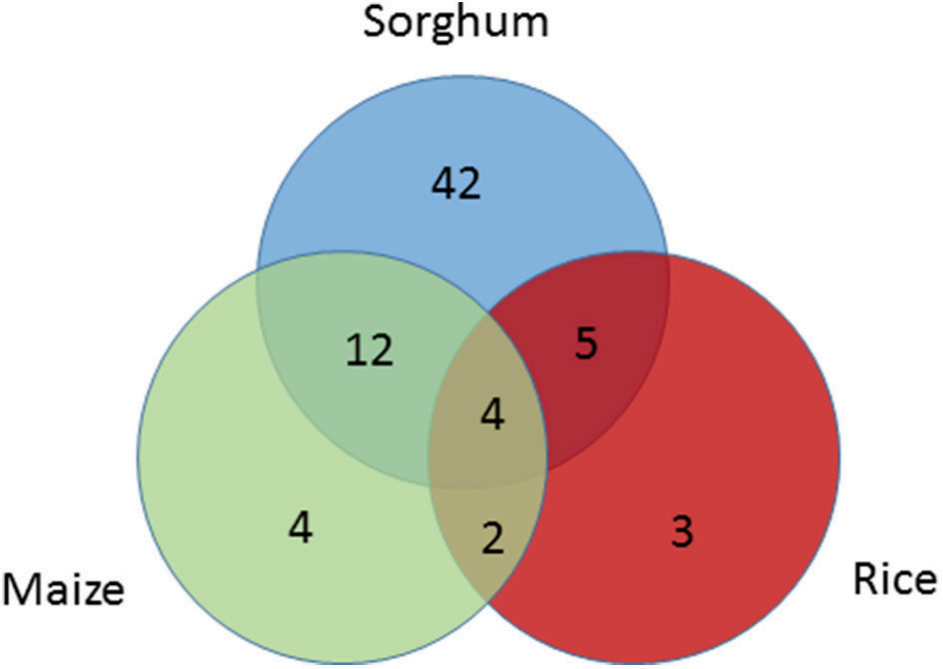
Venn-diagram showing the number seed size genes under selection across species; sorghum (blue), maize (green), and rice (red). Seed size candidate genes under selection in sorghum were identified based on SNP analysis in sorghum, while selection signals on their orthologs in maize and rice were extracted from previous studies.

Seed size genes under selection across species were also identified. Among 15 seed size genes under selection in maize or rice, 12 were also found to be under selection in sorghum based on the SNP level CDS analysis in this study. A broader investigation of parallel domestication selection signals across syntenic orthologs of all the 114 seed size candidate genes in maize (Hufford et al., 2012; Jiao et al., 2012) and rice (He et al., 2011; Huang et al., 2012a; Xu et al., 2012) identified 30 seed size candidate genes in sorghum that have orthologs under selection in maize and/or rice (Table S6). Among these 30 sorghum genes, only one gene was under selection based on the gene level analysis, but 21 genes were identified as being under selection based on the SNP level CDS analysis (Table S6, Figure 3), with 4 of the 9 remaining genes having paralogs under purifying selection in sorghum. The sorghum seed size candidate genes under selection in multiple cereals included Sobic.009G070000 (*GW5* ortholog), Sobic.003G406600 (the of *GW8* ortholog), Sobic.007G101500 (*Bt2* ortholog), Sobic.K041100 (*GIF1* ortholog), and Sobic.005G001500 (*PBF1* ortholog).

## Discussion

Seed size is a typical domestication syndrome trait, with cultivated cereal crops having larger seeds in comparison to their wild progenitors (Doebley et al., 2006). During domestication, large seeded genotypes were selected for their contribution to increased grain yield, but perhaps more importantly also for their positive effect on the quality of end-use products. Utilising the power of whole genome sequencing of diverse sorghum germplasm at the SNP level, combined with comparative genomic analysis of well researched cereal crops such as rice and maize, we identified 114 seed size candidate genes in sorghum. Signatures of domestication were identified in over half (63) of these genes through SNP level analysis of the CDS regions, with a high degree of concordance of seed size candidate genes under selection across species observed. Additionally, a group of seed size candidate genes that exhibited differentially high levels of expression in the seed were found to be more likely under selection during domestication. These results provide new insights into the genetic control of seed size in sorghum and the domestication of the seed size trait in cereal crops. Candidate genes included in this study provide a useful entry point into investigating the genetic factors controlling seed size. An understanding of genetic diversity and evolutionary pressures on these seed size candidate genes in sorghum provides potential targets for manipulating seed size via marker-assist selection or genome editing. In particular, intrinsic seed size genes may prove more amenable to relatively simple interventions in comparison to genes which effect seed size indirectly, for example via grain number.

### Seed size candidate genes under selection are more likely to be intrinsic seed size genes rather than pleiotropic seed size genes

Of the 111 orthologs identified in sorghum based on seed size genes from maize, rice, and *Arabidopsis*, only 9 orthologous genes were identified as being associated with seed size in more than one species (Figure 1). This limited overlap suggests that the sample of seed size genes identified to date in each species is incomplete and/or that the genetic factors influencing seed size vary among species. This is likely to be due to the complexity of the genetic control of seed size, which is controlled by factors involved in intrinsic seed size, such as cell number, cell size, structure and composition, and by physiological factors involved in the carbohydrate supply-demand balance and transport.

Given the differences in plant architecture and physiology across the four species, it seems likely that genes under selection in sorghum that have also been identified as seed size genes in more than one species, either affect intrinsic seed size or directly affect seed number through an effect on panicle architecture, rather than affecting seed size via carbohydrate supply or indirectly affecting seed number. Both situations occurred in this study, as Sobic.001G341700, the ortholog of *GS3* and *ZmGS3* directly influences cell number in the seed, whereas Sobic.002G216600, the ortholog of *DEP1* and *AGG3*, changes panicle branching and therefore seed number (Huang et al., 2009; Mao et al., 2010; Chakravorty et al., 2011; Li S. et al., 2012).

Of the 63 seed size candidate genes identified as being under selection in sorghum, 21 were identified as being under selection in at least one of the other species (Table S6). Genes that exhibited differentially high levels of expression in the seed are more likely to be associated with intrinsic variation for seed size. Our data shows that these genes were much more likely to be under selection during domestication. This provides support for the hypothesis that the modification to seed size during domestication preferentially targeted genes for intrinsic seed size rather than genes that indirectly impact on seed size.

### Base pair level analysis provides a high resolution approach to study domestication signatures on seed size genes

**Figure 4:**
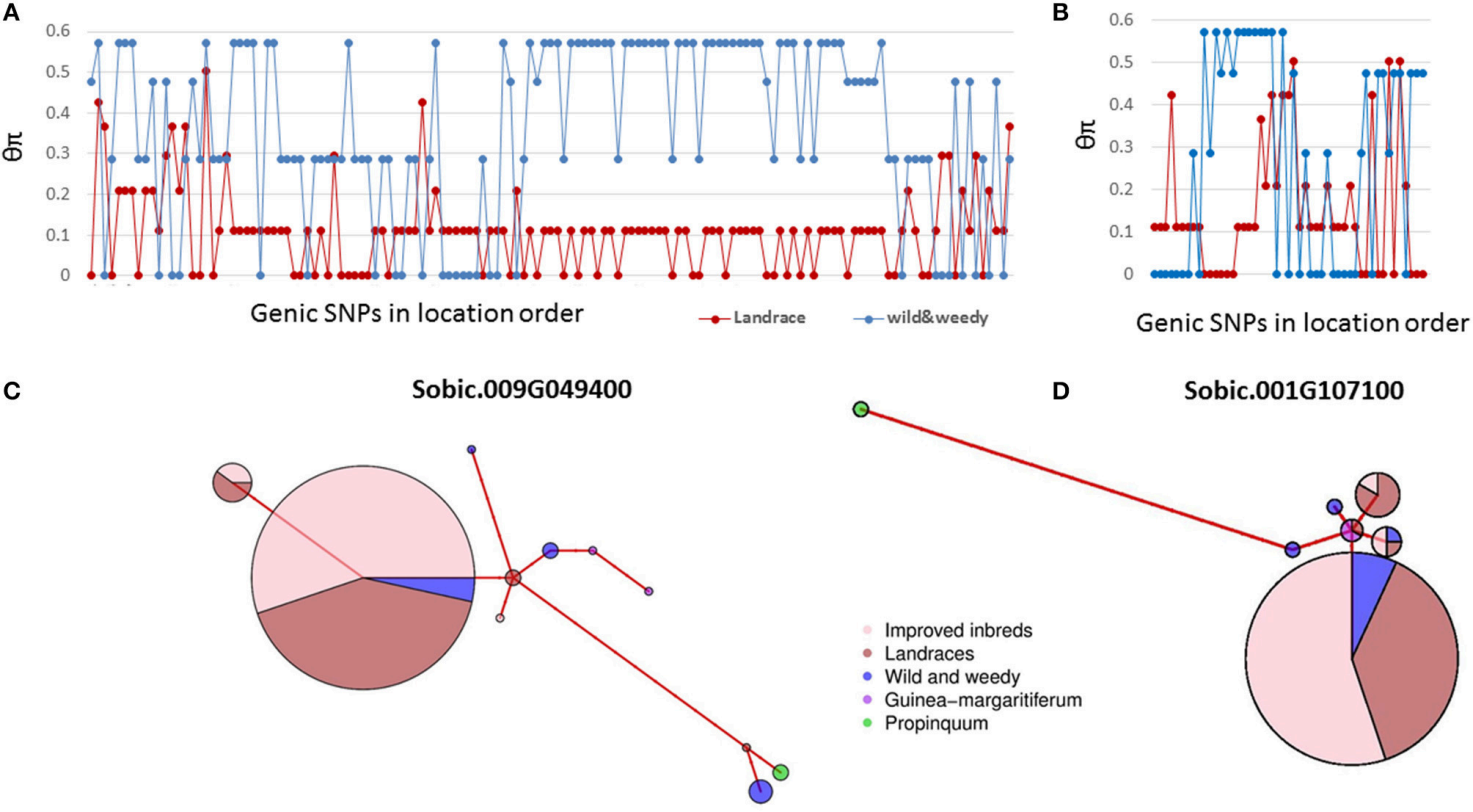
Genetic diversity pattern of two genes under selection. **(A)**, The sequence diversity of SNPs within Sobic.009G049400; **(B)**, The sequence diversity of SNPs within Sobic.001G107100; **(C)**, The haplotype network of Sobic.009G049400; **(D)**, The haplotype network of Sobic.001G107100. Group classification of sorghum accessions used as detailed in Table S2. Colour-coding as follows; improved inbred lines (pink), landraces (red), wild and weedy genotypes (blue), *S. propinquum* (green), and *guinea margaritiferums* (purple). The size of the circles in the haplotype networks is proportionate to the number of accessions with that haplotype. The branch length represents the genetic distance between two haplotypes.

Domestication has shaped sorghum into a productive crop from a wild grass. Previous studies in sorghum have identified thousands of genes underpinning sorghum domestication based on whole genome analyses (Mace et al., 2013; Morris et al., 2013). This study detected selection signals in 63 seed size candidate genes in sorghum identified from cross species analyses based on individual nucleotide level analyses. The nucleotide level analyses provide greater resolution to study domestication signatures than whole gene level rankings. In general, when genes are under strong purifying selection, the gene level analysis may provide sufficient power to identify the signature of selection. For example, in Sobic.009G049400, the ortholog of *SRS3* conferring a round seed phenotype in rice (Kitagawa et al., 2010), 44% of the SNPs were identified with signatures of purifying selection (Figure 4A). The majority of the remaining SNPs in this gene also exhibited the same trend of sequence diversity patterns, resulting in this gene being identified as under purifying selection at both the gene and nucleotide levels (Figure 4C, Mace et al., 2013). However, during domestication, contrasting selections can be imposed on different mutant loci of the same gene (particularly genes with pleiotropic effects) at different times, which results in a gene with chimeric positive and purifying selection signals (Purugganan and Fuller, 2009; Campbell et al., 2016). This situation was observed in this study, where 11 SNPs in the *SRS5* ortholog, Sobic.001G107100, clustering within 50 bp of each other, were identified with signatures of purifying selection (Figure 4B). However, the gene was not identified as being under selection based on the gene level analysis due to the heterogeneous sequence diversity patterns observed across the entire gene length (Figure 4D). In such cases, analyzing each mutant locus separately provides increased resolution to identify the selection signature in comparison to gene level analysis in which contrasting selection signals within the same gene may cancel each other out.

### Common seed size genes under selection across cereals supports parallel domestication of seed size in grass cereals

During crop domestication, human demands have led to a similar suite of traits being changed across a wide range of crops, a phenomenon known as convergent domestication (Lenser and Theißen, 2013). However, whether the same genetic basis underlies parallel changes in different species is still under debate. Early QTL mapping studies found close correspondence of QTLs for seed size, shattering, and flowering time across cereal crops (Paterson et al., 1995), with subsequent detailed QTL analyses identifying high levels of concordance in flowering time QTLs across sorghum and maize (Mace et al., 2013). Recently, *Sh1*, a major QTL controlling shattering, and *HD1*, a major locus conferring flowering time, have been reported to be under parallel selection in multiple cereals (Lin et al., 2012; Liu H. et al., 2015). In this study, among 15 seed size genes previously identified to be under selection in rice or maize, 12 were shown to have orthologs in sorghum under selection during domestication. Genes under parallel selection have been found to be major effect loci of seed size explaining a large proportion of the phenotypic variation (Lenser and Theißen, 2013). The significant overlap of selection signatures on seed size genes in cereals provides support for the role of parallel domestication.

## Conclusions

Seed size and weight are physiologically complex traits controlled by many loci, some of which have been selected during the domestication of cereals. In this study, we have collated a large number of genes controlling seed size and weight across three extensively studied plant model species and identified their sorghum orthologs using comparative genomics analyses. We demonstrated that has domestication in sorghum left signatures of selection genetic signatures on multiple seed size candidate genes. For a number of the seed size genes we found signatures of selection that were common across sorghum, maize and rice, consistent with parallel domestication of the seed size trait. We also found that seed size candidate genes that exhibited differentially high levels of expression in the seed were more likely to be under selection during domestication. Our work sheds light on the processes involved in cereal domestication and provides potential targets for breeding to increase seed size and potentially yield.

## Author contributions

DJ, EM, and IG conceived and designed the experiments: YT, AC, EM, DJ, and XZ collected data; YT, ST, BC, EV, JB, DJ, and EM analyzed data; YT and EM wrote the manuscript. EV, JB, IG, and DJ revised the manuscript. All authors read and approved the final manuscript.

### Conflict of interest statement

The authors declare that the research was conducted in the absence of any commercial or financial relationships that could be construed as a potential conflict of interest.

## Abbreviations

BBH: bidirectional best hit
RoD: reduction of diversity.

## References

[B1] Acreche M. M. Slafer G. A. (2006). Grain weight response to increases in number of grains in wheat in a Mediterranean area. Field Crops Res. 98, 52–59. 10.1016/j.fcr.2005.12.005

[B2] Adamski N. M. Anastasiou E. Eriksson S. O'Neill C. M. Lenhard M. (2009). Local maternal control of seed size by *KLUH/CYP78A5*-dependent growth signaling. Proc. Natl. Acad. Sci. U.S.A. 106, 20115–20120. 10.1073/pnas.0907024106 19892740 PMC2785301

[B3] Alam M. M. Mace E. S. van Oosterom E. J. Cruickshank A. Hunt C. H. Hammer G. L.. (2014). QTL analysis in multiple sorghum populations facilitates the dissection of the genetic and physiological control of tillering. Theor. Appl. Genet. 127, 2253–2266. 10.1007/s00122-014-2377-9 25163934

[B4] Ashikari M. Wu J. Yano M. Sasaki T. Yoshimura A. (1999). Rice gibberellin-insensitive dwarf mutant gene *Dwarf 1* encodes the α-subunit of GTP-binding protein. Proc. Natl. Acad. Sci. U.S.A. 96, 10284–10289. 10.1073/pnas.96.18.10284 10468600 PMC17880

[B5] Beissinger T. M. Hirsch C. N. Vaillancourt B. Deshpande S. Barry K. Buell C. R.. (2014). A genome-wide scan for evidence of selection in a maize population under long-term artificial selection for ear number. Genetics 196, 829–840. 10.1534/genetics.113.160655 24381334 PMC3948809

[B6] Borrell A. K. van Oosterom E. J. Mullet J. E. George-Jaeggli B. Jordan D. R. Klein P. E.. (2014). Stay-green alleles individually enhance grain yield in sorghum under drought by modifying canopy development and water uptake patterns. New Phytol. 203, 817–830. 10.1111/nph.12869 24898064

[B7] Botella J. R. (2012). Can heterotrimeric G proteins help to feed the world? Trends Plant Sci. 17, 563–568. 10.1016/j.tplants.2012.06.002 22748359

[B8] Boyles R. E. Cooper E. A. Myers M. T. Brenton Z. Rauh B. L. Morris G. P.. (2016). Genome-wide association studies of grain yield components in diverse sorghum germplasm. Plant Genome. 9, 1–17. 10.3835/plantgenome2015.09.0091 27898823

[B9] Brugiere N. Humbert S. Rizzo N. Bohn J. Habben J. E. (2008). A member of the maize isopentenyl transferase gene family, *Zea mays* isopentenyl transferase 2 (*ZmIPT2*), encodes a cytokinin biosynthetic enzyme expressed during kernel development. Plant Mol. Biol. 67, 215–229. 10.1007/s11103-008-9312-x 18311542

[B10] Burow G. Xin Z. Hayes C. Burke J. (2014). Characterization of a multiseeded () mutant of sorghum for increasing grain yield. Crop Sci. 54, 2030–2037. 10.2135/cropsci2013.08.0566

[B11] Campbell B. C. Gilding E. K. Mace E. S. Tai S. Tao Y. Prentis P. J.. (2016). Domestication and the storage starch biosynthesis pathway: signatures of selection from a whole sorghum genome sequencing strategy. Plant Biotechnol. J. 14, 2240–2253 10.1111/pbi.12578 27155090 PMC5103234

[B12] Chakravorty D. Trusov Y. Zhang W. Acharya B. R. Sheahan M. B. McCurdy D. W.. (2011). An atypical heterotrimeric G-protein γ-subunit is involved in guard cell K+-channel regulation and morphological development in *Arabidopsis thaliana*. Plant J. 67, 840–851 10.1111/j.1365-313X.2011.04638.x 21575088

[B13] Che R. Tong H. Shi B. Liu Y. Fang S. Liu D.. (2015). Control of grain size and rice yield by GL2-mediated brassinosteroid responses. Nat. Plants 2:15195. 10.1038/nplants.2015.195 27250747

[B14] Chen Y. Xu Y. Luo W. Li W. Chen N. Zhang D.. (2013). The F-box protein *OsFBK12* targets *OsSAMS1* for degradation and affects pleiotropic phenotypes, including leaf senescence, in rice. Plant Physiol. 163, 1673–1685. 10.1104/pp.113.224527 24144792 PMC3850201

[B15] Davidson R. M. Gowda M. Moghe G. Lin H. Vaillancourt B. Shiu S. H.. (2012). Comparative transcriptomics of three Poaceae species reveals patterns of gene expression evolution. Plant J. 71, 492–502. 10.1111/j.1365-313x.2012.05005.x 22443345

[B16] Deng Y. Dong H. Mu J. Ren B. Zheng B. Ji Z.. (2010). Arabidopsis histidine kinase *CKI1* acts upstream of histidine phosphotransfer proteins to regulate female gametophyte development and vegetative growth. Plant Cell 22, 1232–1248. 10.1105/tpc.108.065128 20363773 PMC2879746

[B17] Doebley J. F. Gaut B. S. Smith B. D. (2006). The molecular genetics of crop domestication. Cell 127, 1309–1321. 10.1016/j.cell.2006.12.006 17190597

[B18] Duan P. Rao Y. Zeng D. Yang Y. Xu R. Zhang B.. (2014). *SMALL GRAIN 1*, which encodes a mitogen-activated protein kinase kinase 4, influences grain size in rice. Plant J. 77, 547–557. 10.1111/tpj.12405 24320692

[B19] Eckert C. Offenborn J. N. Heinz T. Armarego-Marriott T. Schültke S. Zhang C.. (2014). The vacuolar calcium sensors *CBL2* and *CBL3* affect seed size and embryonic development in *Arabidopsis thaliana*. Plant J. 78, 146–156. 10.1111/tpj.12456 24479654

[B20] Eom J. S. Cho J. I. Reinders A. Lee S. W. Yoo Y. Tuan P. Q.. (2011). Impaired function of the tonoplast-localized sucrose transporter in rice, *OsSUT2*, limits the transport of vacuolar reserve sucrose and affects plant growth. Plant Physiol. 157, 109–119. 10.1104/pp.111.176982 21771914 PMC3165862

[B21] Fang W. Wang Z. Cui R. Li J. Li Y. (2012). Maternal control of seed size by *EOD3*/*CYP78A6* in *Arabidopsis thaliana*. Plant J. 70, 929–939. 10.1111/j.1365-313X.2012.04907.x 22251317

[B22] Fu F. Xue H. (2010). Coexpression analysis identifies *Rice Starch Regulator1*, a rice *AP2*/*EREBP* family transcription factor, as a novel rice starch biosynthesis regulator. Plant Physiol. 154, 927–938. 10.1104/pp.110.159517 20713616 PMC2949045

[B23] Garcia D. Gerald J. N. F. Berger F. (2005). Maternal control of integument cell elongation and zygotic control of endosperm growth are coordinated to determine seed size in Arabidopsis. Plant Cell, 17, 52–60. 10.1105/tpc.104.027136 15598800 PMC544489

[B24] Hakata M. Kuroda M. Ohsumi A. Hirose T. Nakamura H. Muramatsu M.. (2012). Overexpression of a rice *TIFY* gene increases grain size through enhanced accumulation of carbohydrates in the stem. Biosci. Biotechnol. Biochem. 76, 2129–2134. 10.1271/bbb.120545 23132589

[B25] Hartings H. Maddaloni M. Lazzaroni N. Di Fonzo N. Motto M. Salamini F.. (1989). The *O*_2_ gene which regulates zein deposition in maize endosperm encodes a protein with structural homologies to transcriptional activators. EMBO J. 8, 2795–2801. 2479535 10.1002/j.1460-2075.1989.tb08425.x PMC401325

[B26] He Z. Zhai W. Wen H. Tang T. Wang Y. Lu X.. (2011). Two evolutionary histories in the genome of rice: the roles of domestication genes. PLoS Genet. 7:e1002100. 10.1371/journal.pgen.1002100 21695282 PMC3111475

[B27] Heang D. Sassa H. (2012a). An atypical bHLH protein encoded by *POSITIVE REGULATOR OF GRAIN LENGTH 2* is involved in controlling grain length and weight of rice through interaction with a typical bHLH protein APG. Breed. Sci. 62, 133–141. 10.1270/jsbbs.62.133 23136524 PMC3405970

[B28] Heang D. Sassa H. (2012b). Overexpression of a basic helix–loop–helix gene Antagonist of *PGL1* (*APG*) decreases grain length of rice. Plant Biotechnol. 29, 65–69. 10.5511/plantbiotechnology.12.0117a

[B29] Heinrich G. Francis C. Eastin J. (1983). Stability of grain sorghum yield components across diverse environments. Crop Sci. 23, 209–212. 10.2135/cropsci1983.0011183X002300020004x

[B30] Hong Z. Ueguchi-Tanaka M. Fujioka S. Takatsuto S. Yoshida S. Hasegawa Y.. (2005). The rice brassinosteroid-deficient *dwarf2* mutant, defective in the rice homolog of Arabidopsis *DIMINUTO*/*DWARF1*, is rescued by the endogenously accumulated alternative bioactive brassinosteroid, dolichosterone. Plant Cell 17, 2243–2254. 10.1105/tpc.105.030973 15994910 PMC1182486

[B31] Hong Z. Ueguchi-Tanaka M. Umemura K. Uozu S. Fujioka S. Takatsuto S.. (2003). A rice brassinosteroid-deficient mutant, ebisu dwarf (*d2*), is caused by a loss of function of a new member of cytochrome P450. Plant Cell, 15, 2900–2910. 10.1105/tpc.014712 14615594 PMC282825

[B32] Hu J. Wang Y. Fang Y. Zeng L. Xu J. Yu H.. (2015). A rare allele of *GS2* enhances grain size and grain yield in rice. Mol. Plant 8, 1455–1465. 10.1016/j.molp.2015.07.002 26187814

[B33] Hu X. Qian Q. Xu T. Zhang Y. E. Dong G. Gao T.. (2013). The U-box E3 ubiquitin ligase *TUD1* functions with a heterotrimeric G α subunit to regulate brassinosteroid-mediated growth in rice. PLoS Genet. 9:e1003391. 10.1371/journal.pgen.1003391 23526892 PMC3597501

[B34] Huang X. Kurata N. Wei X. Wang Z. Wang A. Zhao Q.. (2012a). A map of rice genome variation reveals the origin of cultivated rice. Nature 490, 497–501. 10.1038/nature11532 23034647 PMC7518720

[B35] Huang X. Qian Q. Liu Z. Sun H. He S. Luo D.. (2009). Natural variation at the *DEP1* locus enhances grain yield in rice. Nat. Genet. 41, 494–497. 10.1038/ng.352 19305410

[B36] Huang X. Zhao Y. Wei X. Li C. Wang A. Zhao Q.. (2012b). Genome-wide association study of flowering time and grain yield traits in a worldwide collection of rice germplasm. Nat. Genet. 44, 32–39. 10.1038/ng.1018 22138690

[B37] Hudson R. R. Slatkin M. Maddison W. P. (1992). Estimation of levels of gene flow from DNA sequence data. Genetics 132, 583–589. 1427045 10.1093/genetics/132.2.583 PMC1205159

[B38] Hufford M. B. Xu X. Van Heerwaarden J. Pyhäjärvi T. Chia J. M. Cartwright R. A.. (2012). Comparative population genomics of maize domestication and improvement. Nat. Genet. 44, 808–811. 10.1038/ng.2309 22660546 PMC5531767

[B39] Hutchison C. E. Li J. Argueso C. Gonzalez M. Lee E. Lewis M. W.. (2006). The Arabidopsis histidine phosphotransfer proteins are redundant positive regulators of cytokinin signaling. Plant Cell 18, 3073–3087. 10.1105/tpc.106.045674 17122069 PMC1693944

[B40] Ishimaru K. Hirotsu N. Madoka Y. Murakami N. Hara N. Onodera H.. (2013). Loss of function of the IAA-glucose hydrolase gene *TGW6* enhances rice grain weight and increases yield. Nat. Genet. 45, 707–711. 10.1038/ng.2612 23583977

[B41] Jagadish K. S. Kishor P. B. K. Bahuguna R. N. von Wirén N. Sreenivasulu N. (2015). Staying alive or going to die during terminal senescence-an enigma surrounding yield stability. Front. Plant Sci. 6:01070. 10.3389/fpls.2015.01070 26648957 PMC4663250

[B42] Jakobsson A. Eriksson O. (2000). A comparative study of seed number, seed size, seedling size and recruitment in grassland plants. Oikos 88, 494–502. 10.1034/j.1600-0706.2000.880304.x

[B43] Jenner C. (1994). Starch synthesis in the kernel of wheat under high temperature conditions. Funct. Plant Biol. 21, 791–806. 10.1071/pp9940791

[B44] Jiang L. Yu X. Qi X. Yu Q. Deng S. Bai B.. (2013). Multigene engineering of starch biosynthesis in maize endosperm increases the total starch content and the proportion of amylose. Transgenic Res. 22, 1133–1142. 10.1007/s11248-013-9717-4 23740205

[B45] Jiang W. Huang H. Hu Y. Zhu S. Wang Z. Lin W. (2013). Brassinosteroid regulates seed size and shape in Arabidopsis. Plant Physiol. 162, 1965–1977. 10.1104/pp.113.217703 23771896 PMC3729775

[B46] Jiao Y. Zhao H. Ren L. Song W. Zeng B. Guo J.. (2012). Genome-wide genetic changes during modern breeding of maize. Nat. Genet. 44, 812–815. 10.1038/ng.2312 22660547

[B47] Kitagawa K. Kurinami S. Oki K. Abe Y. Ando T. Kono I.. (2010). A novel kinesin 13 protein regulating rice seed length. Plant Cell Physiol. 51, 1315–1329. 10.1093/pcp/pcq092 20587735

[B48] Kondou Y. Nakazawa M. Kawashima M. Ichikawa T. Yoshizumi T. Suzuki K.. (2008). *RETARDED GROWTH OF EMBRYO1*, a new basic helix-loop-helix protein, expresses in endosperm to control embryo growth. Plant Physiol. 147, 1924–1935. 10.1104/pp.108.118364 18567831 PMC2492639

[B49] Lang Z. Wills D. M. Lemmon Z. H. Shannon L. M. Bukowski R. Wu Y.. (2014). Defining the role of *prolamin-box binding factor1* gene during maize domestication. J. Hered. 105, 576–582. 10.1093/jhered/esu019 24683184 PMC6373496

[B50] Lee W. Pedersen J. F. Shelton D. (2002). Relationship of sorghum kernel size to physiochemical, milling, pasting, and cooking properties. Food Res. Int. 35, 643–649. 10.1016/S0963-9969(01)00167-3

[B51] Lenser T. Theißen G. (2013). Molecular mechanisms involved in convergent crop domestication. Trends Plant Sci. 18, 704–714. 10.1016/j.tplants.2013.08.007 24035234

[B52] Li F. Liu W. Tang J. Chen J. Tong H. Hu B.. (2010). Rice *DENSE AND ERECT PANICLE 2* is essential for determining panicle outgrowth and elongation. Cell Res. 20, 838–849. 10.1038/cr.2010.69 20502443

[B53] Li J. Chu H. Zhang Y. Mou T. Wu C. Zhang Q.. (2012). The rice *HGW* gene encodes a ubiquitin-associated (UBA) domain protein that regulates heading date and grain weight. PLoS ONE 7:e34231. 10.1371/journal.pone.0034231 22457828 PMC3311617

[B54] Li J. Nie X. Tan J. L. H. Berger F. (2013). Integration of epigenetic and genetic controls of seed size by cytokinin in Arabidopsis. Proc. Natl Acad. Sci. U.S.A. 110, 15479–15484. 10.1073/pnas.1305175110 24003120 PMC3780859

[B55] Li Q. Li L. Yang X. Warburton M. L. Bai G. Dai J.. (2010a). Relationship, evolutionary fate and function of two maize co-orthologs of rice *GW2* associated with kernel size and weight. BMC Plant Biol. 10:143. 10.1186/1471-2229-10-143 20626916 PMC3017803

[B56] Li Q. Yang X. Bai G. Warburton M. L. Mahuku G. Gore M.. (2010b). Cloning and characterization of a putative *GS3* ortholog involved in maize kernel development. Theor. Appl. Genet. 120, 753–763. 10.1007/s00122-009-1196-x 19898828

[B57] Li S. Liu Y. Zheng L. Chen L. Li N. Corke F.. (2012). The plant-specific G protein γ subunit *AGG3* influences organ size and shape in *Arabidopsis thaliana*. New Phytol. 194, 690–703. 10.1111/j.1469-8137.2012.04083.x 22380792

[B58] Li X. J. Zhang Y. F. Hou M. Sun F. Shen Y. Xiu Z. H.. (2014). *Small kernel 1* encodes a pentatricopeptide repeat protein required for mitochondrial nad7 transcript editing and seed development in maize (*Zea mays*) and rice (*Oryza sativa*). Plant J. 79, 797–809. 10.1111/tpj.12584 24923534

[B59] Li X. Sun L. Tan L. Liu F. Zhu Z. Fu Y.. (2012). *TH1*, a DUF640 domain-like gene controls lemma and palea development in rice. Plant Mol. Biol. 78, 351–359. 10.1007/s11103-011-9868-8 22203474

[B60] Li Y. Fan C. Xing Y. Jiang Y. Luo L. Sun L.. (2011). Natural variation in *GS5* plays an important role in regulating grain size and yield in rice. Nat. Genet. 43, 1266–1269. 10.1038/ng.977 22019783

[B61] Li Y. Zheng L. Corke F. Smith C. Bevan M. W. (2008). Control of final seed and organ size by the *DA1* gene family in *Arabidopsis thaliana*. Genes Dev. 22, 1331–1336. 10.1101/gad.463608 18483219 PMC2377187

[B62] Lin Z. Li X. Shannon L. M. Yeh C.-T. Wang M. L. Bai G.. (2012). Parallel domestication of the *Shattering1* genes in cereals. Nat. Genet. 44, 720–724. 10.1038/ng.2281 22581231 PMC3532051

[B63] Liu H. Liu H. Zhou L. Zhang Z. Zhang X. Wang M.. (2015). Parallel domestication of the *heading date 1* gene in cereals. Mol. Biol. Evol. 32, 2726–2737. 10.1093/molbev/msv148 26116860

[B64] Liu J. Chen J. Zheng X. Wu F. Lin Q. Heng Y.. (2017). GW5 acts in the brassinosteroid signalling pathway to regulate grain width and weight in rice. Nat. Plants 3:17043. 10.1038/nplants.2017.43 28394310

[B65] Liu L. Tong H. Xiao Y. Che R. Xu F. Hu B.. (2015). Activation of *Big Grain1* significantly improves grain size by regulating auxin transport in rice. Proc. Natl. Acad. Sci. U.S.A. 112, 11102–11107. 10.1073/pnas.1512748112 26283354 PMC4568269

[B66] Luo J. Liu H. Zhou T. Gu B. Huang X. Shangguan Y.. (2013). *An-1* encodes a basic helix-loop-helix protein that regulates awn development, grain size, and grain number in rice. Plant Cell 25, 3360–3376. 10.1105/tpc.113.113589 24076974 PMC3809537

[B67] Luo M. Bilodeau P. Dennis E. S. Peacock W. J. Chaudhury A. (2000). Expression and parent-of-origin effects for *FIS2, MEA*, and *FIE* in the endosperm and embryo of developing Arabidopsis seeds. Proc. Natl. Acad. Sci. U.S.A. 97, 10637–10642. 10.1073/pnas.170292997 10962025 PMC27077

[B68] Luo M. Dennis E. S. Berger F. Peacock W. J. Chaudhury A. (2005). *MINISEED3* (*MINI3*), a WRKY family gene, and *HAIKU2* (*IKU2*), a leucine-rich repeat (LRR) KINASE gene, are regulators of seed size in Arabidopsis. Proc. Natl. Acad. Sci. U.S.A. 102, 17531–17536. 10.1073/pnas.0508418102 16293693 PMC1297679

[B69] Ma B. He S. Duan K. Yin C. Chen H. Yang C.. (2013). Identification of rice ethylene-response mutants and characterization of *MHZ7*/*OsEIN2* in distinct ethylene response and yield trait regulation. Mol. Plant 6, 1830–1848. 10.1093/mp/sst087 23718947

[B70] Mace E. S. Tai S. Gilding E. K. Li Y. Prentis P. J. Bian L.. (2013). Whole-genome sequencing reveals untapped genetic potential in Africa's indigenous cereal crop sorghum. Nat. Commun. 4:2320. 10.1038/ncomms3320 23982223 PMC3759062

[B71] Manga V. K. Yadav O. P. (1995). Effect of seed size on development traits and ability to tolerate drought in pearl millet. J. Arid Environ. 29, 169–172. 10.1016/S0140-1963(05)80087-4

[B72] Mao H. Sun S. Yao J. Wang C. Yu S. Xu C.. (2010). Linking differential domain functions of the *GS3* protein to natural variation of grain size in rice. Proc. Natl. Acad. Sci. U.S.A. 107, 19579–19584. 10.1073/pnas.1014419107 20974950 PMC2984220

[B73] Martin A. Lee J. Kichey T. Gerentes D. Zivy M. Tatout C.. (2006). Two cytosolic glutamine synthetase isoforms of maize are specifically involved in the control of grain production. Plant Cell 18, 3252–3274. 10.1105/tpc.106.042689 17138698 PMC1693956

[B74] Miller M. E. Chourey P. S. (1992). The maize invertase-deficient miniature-1 seed mutation is associated with aberrant pedicel and endosperm development. Plant Cell 4, 297–305. 10.1105/tpc.4.3.297 12297647 PMC160130

[B75] Mizukami Y. Fischer R. L. (2000). Plant organ size control: *AINTEGUMENTA* regulates growth and cell numbers during organogenesis. Proc. Natl. Acad. Sci. U.S.A. 97, 942–947. 10.1073/pnas.97.2.942 10639184 PMC15435

[B76] Moles A. T. Westoby M. (2004). Seedling survival and seed size: a synthesis of the literature. J. Ecol. 92, 372–383. 10.1111/j.0022-0477.2004.00884.x

[B77] Mori M. Nomura T. Ooka H. Ishizaka M. Yokota T. Sugimoto K.. (2002). Isolation and characterization of a rice dwarf mutant with a defect in brassinosteroid biosynthesis. Plant Physiol. 130, 1152–1161. 10.1104/pp.007179 12427982 PMC166636

[B78] Morinaka Y. Sakamoto T. Inukai Y. Agetsuma M. Kitano H. Ashikari M.. (2006). Morphological alteration caused by brassinosteroid insensitivity increases the biomass and grain production of rice. Plant Physiol. 141, 924–931. 10.1104/pp.106.077081 16714407 PMC1489896

[B79] Morris G. P. Ramu P. Deshpande S. P. Hash C. T. Shah T. Upadhyaya H. D.. (2013). Population genomic and genome-wide association studies of agroclimatic traits in sorghum. Proc. Natl. Acad. Sci. U.S.A. 110, 453–458. 10.1073/pnas.1215985110 23267105 PMC3545811

[B80] Na J. K. Seo M. H. Yoon I. S. Lee Y. H. Lee K. O. Kim D. Y. (2012). Involvement of rice Polycomb protein *OsFIE2* in plant growth and seed size. Plant Biotechnol. Rep. 6, 339–346. 10.1007/s11816-012-0229-0

[B81] Nakagawa H. Tanaka A. Tanabata T. Ohtake M. Fujioka S. Nakamura H.. (2012). *Short grain1* decreases organ elongation and brassinosteroid response in rice. Plant Physiol. 158, 1208–1219. 10.1104/pp.111.187567 22209874 PMC3291246

[B82] Nei M. Li W. H. (1979). Mathematical model for studying genetic variation in terms of restriction endonucleases. Proc. Natl. Acad. Sci. U.S.A. 76, 5269–5273. 10.1073/pnas.76.10.5269 291943 PMC413122

[B83] Ohto M. A. Floyd S. K. Fischer R. L. Goldberg R. B. Harada J. J. (2009). Effects of *APETALA2* on embryo, endosperm, and seed coat development determine seed size in Arabidopsis. Sex. Plant Reprod. 22, 277–289. 10.1007/s00497-009-0116-1 20033449 PMC2796121

[B84] Okushima Y. Mitina I. Quach H. L. Theologis A. (2005). *AUXIN RESPONSE FACTOR 2* (*ARF2*): a pleiotropic developmental regulator. Plant J. 43, 29–46. 10.1111/j.1365-313X.2005.02426.x 15960614

[B85] Paradis E. (2010). pegas: an R package for population genetics with an integrated–modular approach. Bioinformatics 26, 419–420. 10.1093/bioinformatics/btp696 20080509

[B86] Paradis E. Claude J. Strimmer K. (2004). APE: analyses of phylogenetics and evolution in R language. Bioinformatics 20, 289–290. 10.1093/bioinformatics/btg412 14734327

[B87] Paterson A. H. Lin Y. R. Li Z. Schertz K. F. (1995). Convergent domestication of cereal crops by independent mutations at corresponding genetic loci. Science 269, 1714–1718. 17821643 10.1126/science.269.5231.1714

[B88] Peltonen-Sainio P. Kangas A. Salo Y. Jauhiainen L. (2007). Grain number dominates grain weight in temperate cereal yield determination: evidence based on 30 years of multi-location trials. Field Crops Res. 100, 179–188. 10.1016/j.fcr.2006.07.002

[B89] Pfeifer B. Wittelsbürger U. Onsins S. E. R. Lercher M. J. (2014). PopGenome: an efficient Swiss army knife for population genomic analyses in R. Mol. Biol. Evol. 31, 1929–1936. 10.1093/molbev/msu136 24739305 PMC4069620

[B90] Purugganan M. D. Fuller D. Q. (2009). The nature of selection during plant domestication. Nature 457, 843–848. 10.1038/nature07895 19212403

[B91] Qi P. Lin Y. Song X. Shen J. Huang W. Shan J.. (2012). The novel quantitative trait locus *GL3. 1* controls rice grain size and yield by regulating Cyclin-T1; 3. Cell Res. 22, 1666–1680. 10.1038/cr.2012.151 23147796 PMC3515756

[B92] Qiao Y. Piao R. Shi J. Lee S. I. Jiang W. Kim B. K.. (2011). Fine mapping and candidate gene analysis of dense and erect panicle 3, *DEP3*, which confers high grain yield in rice (*Oryza sativa* L.). Theor. Appl. Genet. 122, 1439–1449. 10.1007/s00122-011-1543-6 21318372

[B93] Riefler M. Novak O. Strnad M. Schmülling T. (2006). Arabidopsis cytokinin receptor mutants reveal functions in shoot growth, leaf senescence, seed size, germination, root development, and cytokinin metabolism. Plant Cell 18, 40–54. 10.1105/tpc.105.037796 16361392 PMC1323483

[B94] Sadras V. O. (2007). Evolutionary aspects of the trade-off between seed size and number in crops. Field Crops Res. 100, 125–138. 10.1016/j.fcr.2006.07.004

[B95] Sands D. C. Morris C. E. Dratz E. A. Pilgeram A. L. (2009). Elevating optimal human nutrition to a central goal of plant breeding and production of plant-based foods. Plant Sci. 177, 377–389. 10.1016/j.plantsci.2009.07.011 20467463 PMC2866137

[B96] Schmidt R. Mieulet D. Hubberten H. M. Obata T. Hoefgen R. Fernie A. R.. (2013). *SALT-RESPONSIVE ERF1* regulates reactive oxygen species–dependent signaling during the initial response to salt stress in rice. Plant Cell 25, 2115–2131. 10.1105/tpc.113.113068 23800963 PMC3723616

[B97] Segami S. Kono I. Ando T. Yano M. Kitano H. Miura K.. (2012). *Small and round seed 5* gene encodes alpha-tubulin regulating seed cell elongation in rice. Rice 5, 1–10. 10.1186/1939-8433-5-4 24764504 PMC3834490

[B98] Shannon J. C. Pien F. M. Cao H. Liu K. (1998). *Brittle-1*, an adenylate translocator, facilitates transfer of extraplastidial synthesized ADP-glucose into amyloplasts of maize endosperms. Plant Physiol. 117, 1235–1252. 10.1104/pp.117.4.1235 9701580 PMC34888

[B99] She K. C. Kusano H. Koizumi K. Yamakawa H. Hakata M. Imamura T.. (2010). A novel factor *FLOURY ENDOSPERM2* is involved in regulation of rice grain size and starch quality. Plant Cell 22, 3280–3294. 10.1105/tpc.109.070821 20889913 PMC2990130

[B100] Shure M. Wessler S. Fedoroff N. (1983). Molecular identification and isolation of the Waxy locus in maize. Cell 35, 225–233. 10.1016/0092-8674(83)90225-8 6313224

[B101] Si L. Chen J. Huang X. Gong H. Luo J. Hou Q.. (2016). *OsSPL13* controls grain size in cultivated rice. Nat. Genet. 48, 447–456 10.1038/ng.3518 26950093

[B102] Song X. J. Kuroha T. Ayano M. Furuta T. Nagai K. Komeda N.. (2015). Rare allele of a previously unidentified histone H4 acetyltransferase enhances grain weight, yield, and plant biomass in rice. Proc. Natl. Acad. Sci. U.S.A. 112, 76–81. 10.1073/pnas.1421127112 25535376 PMC4291654

[B103] Song X. Huang W. Shi M. Zhu M. Lin H. (2007). A QTL for rice grain width and weight encodes a previously unknown RING-type E3 ubiquitin ligase. Nat. Genet. 39, 623–630. 10.1038/ng2014 17417637

[B104] Sosso D. Luo D. Li Q. B. Sasse J. Yang J. Gendrot G.. (2015). Seed filling in domesticated maize and rice depends on SWEET-mediated hexose transport. Nat. Genet. 47, 1489–1493. 10.1038/ng.3422 26523777

[B105] Su'udi M. Cha J. Y. Ahn I. P. Kwak Y. S. Woo Y. M. Son D. (2012). Functional characterization of a B-type cell cycle switch 52 in rice (*OsCCS52B*). Plant Cell Tissue Organ Cult. 111, 101–111. 10.1007/s11240-012-0176-z

[B106] Sui P. Jin J. Ye S. Mu C. Gao J. Feng H.. (2012). H3K36 methylation is critical for brassinosteroid-regulated plant growth and development in rice. Plant J. 70, 340–347. 10.1111/j.1365-313X.2011.04873.x 22136623

[B107] Tajima F. (1989). Statistical method for testing the neutral mutation hypothesis by DNA polymorphism. Genetics 123, 585–595. 2513255 10.1093/genetics/123.3.585 PMC1203831

[B108] Takano-Kai N. Jiang H. Kubo T. Sweeney M. Matsumoto T. Kanamori H.. (2009). Evolutionary history of GS3, a gene conferring grain length in rice. Genetics 182, 1323–1334. 10.1534/genetics.109.103002 19506305 PMC2728869

[B109] Tanabe S. Ashikari M. Fujioka S. Takatsuto S. Yoshida S. Yano M.. (2005). A novel cytochrome P450 is implicated in brassinosteroid biosynthesis via the characterization of a rice dwarf mutant, *dwarf11*, with reduced seed length. Plant Cell 17, 776–790. 10.1105/tpc.104.024950 15705958 PMC1069698

[B110] Wang A. Garcia D. Zhang H. Feng K. Chaudhury A. Berger F.. (2010). The VQ motif protein *IKU1* regulates endosperm growth and seed size in Arabidopsis. Plant J. 63, 670–679. 10.1111/j.1365-313X.2010.04271.x 20545893

[B111] Wang E. Wang J. Zhu X. Hao W. Wang L. Li Q.. (2008). Control of rice grain-filling and yield by a gene with a potential signature of domestication. Nat. Genet. 40, 1370–1374. 10.1038/ng.220 18820698

[B112] Wang G. Wang F. Wang G. Wang F. Zhang X. Zhong M.. (2012). *Opaque1* encodes a myosin XI motor protein that is required for endoplasmic reticulum motility and protein body formation in maize endosperm. Plant Cell 24, 3447–3462. 10.1105/tpc.112.101360 22892319 PMC3462643

[B113] Wang S. Li S. Liu Q. Wu K. Zhang J. Wang S.. (2015). The *OsSPL16-GW7* regulatory module determines grain shape and simultaneously improves rice yield and grain quality. Nat. Genet. 47, 949–954. 10.1038/ng.3352 26147620

[B114] Wang S. Wu K. Yuan Q. Liu X. Liu Z. Lin X.. (2012). Control of grain size, shape and quality by *OsSPL16* in rice. Nat. Genet. 44, 950–954. 10.1038/ng.2327 22729225

[B115] Wang Y. Xiong G. Hu J. Jiang L. Yu H. Xu J.. (2015). Copy number variation at the *GL7* locus contributes to grain size diversity in rice. Nat. Genet. 47, 944–948. 10.1038/ng.3346 26147619

[B116] Weng J. Li B. Liu C. Yang X. Wang H. Hao Z.. (2013). A non-synonymous SNP within the *isopentenyl transferase 2* locus is associated with kernel weight in Chinese maize inbreds (*Zea mays L*.). BMC Plant Biol. 13:98. 10.1186/1471-2229-13-98 23826856 PMC3704264

[B117] Westoby M. Jurado E. Leishman M. (1992). Comparative evolutionary ecology of seed size. Trends Ecol. Evol. 7, 368–372. 10.1016/0169-5347(92)90006-W 21236070

[B118] Whitt S. R. Wilson L. M. Tenaillon M. I. Gaut B. S. Buckler E. S. (2002). Genetic diversity and selection in the maize starch pathway. Proc. Natl. Acad. Sci. U.S.A. 99, 12959–12962. 10.1073/pnas.202476999 12244216 PMC130568

[B119] Wills D. M. Whipple C. J. Takuno S. Kursel L. E. Shannon L. M. Ross-Ibarra J.. (2013). From many, one: genetic control of prolificacy during maize domestication. PLoS Genet. 9:e1003604. 10.1371/journal.pgen.1003604 23825971 PMC3694832

[B120] Wisser R. J. Murray S. C. Kolkman J. M. Ceballos H. Nelson R. J. (2008). Selection mapping of loci for quantitative disease resistance in a diverse maize population. Genetics 180, 583–599. 10.1534/genetics.108.090118 18723892 PMC2535707

[B121] Wolf Y. I. Koonin E. V. (2012). A tight link between orthologs and bidirectional best hits in bacterial and archaeal genomes. Genome Bio Evol. 4, 1286–1294. 10.1093/gbe/evs100 23160176 PMC3542571

[B122] Wright S. I. Charlesworth B. (2004). The HKA test revisited. Genetics 168, 1071–1076. 10.1534/genetics.104.026500 15514076 PMC1448833

[B123] Wu C. Trieu A. Radhakrishnan P. Kwok S. F. Harris S. Zhang K.. (2008). Brassinosteroids regulate grain filling in rice. Plant Cell 20, 2130–2145. 10.1105/tpc.107.055087 18708477 PMC2553602

[B124] Xiao W. Brown R. C. Lemmon B. E. Harada J. J. Goldberg R. B. Fischer R. L. (2006). Regulation of seed size by hypomethylation of maternal and paternal genomes. Plant Physiol. 142, 1160–1168. 10.1104/pp.106.088849 17012404 PMC1630758

[B125] Xing Y. Zhang Q. (2010). Genetic and molecular bases of rice yield. Annu. Rev. Plant Biol. 61, 421–442. 10.1146/annurev-arplant-042809-112209 20192739

[B126] Xu F. Fang J. Ou S. Gao S. Zhang F. Du L.. (2015). Variations in *CYP78A13* coding region influence grain size and yield in rice. Plant Cell Environ. 38, 800–811. 10.1111/pce.12452 25255828

[B127] Xu X. Liu X. Ge S. Jensen J. D. Hu F. Li X.. (2012). Resequencing 50 accessions of cultivated and wild rice yields markers for identifying agronomically important genes. Nat. Biotechnol. 30, 105–111. 10.1038/nbt.2050 22158310

[B128] Yang Z. van Oosterom E. J. Jordan D. R. Hammer G. L. (2009). Pre-anthesis ovary development determines genotypic differences in potential kernel weight in sorghum. J Exp Bot. 60, 1399–1408. 10.1093/jxb/erp019 19228817 PMC2657540

[B129] Yang Z. van Oosterom E. J. Jordan D. R. Doherty A. Hammer G. L. (2010). Genetic variation in potential kernel size affects kernel growth and yield of sorghum. Crop Sci. 50, 685–695. 10.2135/cropsci2009.06.0294

[B130] Yoine M. Nishii T. Nakamura K. (2006). *Arabidopsis* UPF1 RNA helicase for nonsense-mediated mRNA decay is involved in seed size control and is essential for growth. Plant Cell Physiol. 47, 572–580. 10.1093/pcp/pcj035 16540482

[B131] Yu F. Li J. Huang Y. Liu L. Li D. Chen L.. (2014). FERONIA receptor kinase controls seed size in Arabidopsis thaliana. Mol. Plant 7, 920–922. 10.1093/mp/ssu010 24482438

[B132] Zhang B. Liu X. Qian Q. Liu L. Dong G. Xiong G.. (2011). Golgi nucleotide sugar transporter modulates cell wall biosynthesis and plant growth in rice. Proc. Natl. Acad. Sci. U.S.A. 108, 5110–5115. 10.1073/pnas.1016144108 21383162 PMC3064376

[B133] Zhang D. Li J. Compton R. O. Robertson J. Goff V. H. Epps E.. (2015). Comparative genetics of seed size traits in divergent cereal lineages represented by sorghum (Panicoidae) and rice (Oryzoidae). G3: Genes Genom. Genet 5, 1117–1128. 10.1534/genetics.115.177170 25834216 PMC4478542

[B134] Zhang X. Wang J. Huang J. Lan H. Wang C. Yin C.. (2012). Rare allele of *OsPPKL1* associated with grain length causes extra-large grain and a significant yield increase in rice. Proc. Natl. Acad. Sci. U.S.A. 109, 21534–21539. 10.1073/pnas.1219776110 23236132 PMC3535600

[B135] Zolkevich V. Prusakova L. Lizandr A. (1958). Translocation of assimilates and respiration of conductive tissues in relation to soil moisture. Fiziologiya Rastenii. 5, 337–344.

[B136] Zuo J. Li J. (2014). Molecular genetic dissection of quantitative trait loci regulating rice grain size. Annu. Rev. Genet. 48, 99–118. 10.1146/annurev-genet-120213-092138 25149369

